# Development and performance assessment of novel machine learning models for predicting postoperative pneumonia in aneurysmal subarachnoid hemorrhage patients: external validation in MIMIC-IV

**DOI:** 10.3389/fneur.2024.1341252

**Published:** 2024-04-15

**Authors:** Xinbo Li, Chengwei Zhang, Jiale Wang, Chengxing Ye, Jiaqian Zhu, Qichuan Zhuge

**Affiliations:** ^1^Department of Neurosurgery, The First Affiliated Hospital of Wenzhou Medical University, Wenzhou, China; ^2^Wenzhou Medical University, Wenzhou, China

**Keywords:** aneurysmal subarachnoid hemorrhage, postoperative pneumonia, machine learning, endovascular treatment, prediction

## Abstract

**Background:**

Postoperative pneumonia (POP) is one of the primary complications after aneurysmal subarachnoid hemorrhage (aSAH) and is associated with postoperative mortality, extended hospital stay, and increased medical fee. Early identification of pneumonia and more aggressive treatment can improve patient outcomes. We aimed to develop a model to predict POP in aSAH patients using machine learning (ML) methods.

**Methods:**

This internal cohort study included 706 patients with aSAH undergoing intracranial aneurysm embolization or aneurysm clipping. The cohort was randomly split into a train set (80%) and a testing set (20%). Perioperative information was collected from participants to establish 6 machine learning models for predicting POP after surgical treatment. The area under the receiver operating characteristic curve (AUC), precision-recall curve were used to assess the accuracy, discriminative power, and clinical validity of the predictions. The final model was validated using an external validation set of 97 samples from the Medical Information Mart for Intensive Care IV (MIMIC-IV) database.

**Results:**

In this study, 15.01% of patients in the training set and 12.06% in the testing set with POP after underwent surgery. Multivariate logistic regression analysis showed that mechanical ventilation time (MVT), Glasgow Coma Scale (GCS), Smoking history, albumin level, neutrophil-to-albumin Ratio (NAR), c-reactive protein (CRP)-to-albumin ratio (CAR) were independent predictors of POP. The logistic regression (LR) model presented significantly better predictive performance (AUC: 0.91) than other models and also performed well in the external validation set (AUC: 0.89).

**Conclusion:**

A machine learning model for predicting POP in aSAH patients was successfully developed using a machine learning algorithm based on six perioperative variables, which could guide high-risk POP patients to take appropriate preventive measures.

## Introduction

Aneurysmal subarachnoid hemorrhage (aSAH), primarily caused by the rupture of intracranial aneurysms, is a devastating disease with high morbidity and mortality (1, 2). The total occurrence of aSAH is about 9.1 per 100,000 people, accounts for 2–7% of all strokes (3, 4). Despite the advances in the treatment of aneurysmal subarachnoid hemorrhage (aSAH), such as endovascular coil embolization and microsurgery, postoperative complications can significantly impact the prognosis of patients, prolonged the time of hospitalization and lead to increased economic costs (2, 5).

Among these complications, postoperative pneumonia (POP), which occurs in 10–30% aSAH patients after surgical treatment, is considered a critical complication closely associated with the prognosis of aSAH patients (2, 6–10). Our previous study have found a close relationship between smoking history, delayed cerebral ischemia (DCI), mechanical ventilation time (MVT), Glasgow Coma Scale (GCS), Albumin, c-reactive protein (CRP). Using these variables, our team initially constructed a nomogram model to predicat POP for aSAH patients (11). But with the further research, numerous scholars contend many composite indicators were very effective for postoperative complications prediction, such as neutrophil-to-lymphocyte Ratio (NLR), prognostic nutrition index (PNI), neutrophil-to-albumin Ratio (NAR), CRP-to-albumin ratio (CAR), D-Dimer-to-Albumin Ratio (DAR) (2, 5, 12, 13). The aforementioned indicators, however, were not incorporated as variables in our previous study. The evaluation of the validity of these variables was therefore deemed necessary in order to ascertain their potential for enhancing the predictive power of our model.

In addition, although previous studies have suggested that conventional LR can provide a clinical prediction model that is easy to interpret, when conventional LR is used for complex multivariate non-linear relationships, complex transformations are often required owing to low robustness and multicollinearity between variables (14). Therefore, recent work has highlighted the potential of machine learning (ML) algorithms for stroke-related complications in stroke patients (15, 16). Savarraj et al. suggested that ML models significantly outperform conventional LR in predicting functional outcomes and has the potential to improve SAH management (17). The ML models possess the potential to outperform conventional linear or logistic regression models due to their exceptional ability in identifying intricate and nonlinear relationships among a multitude of prognostic variables (16). Thus, in this study, we conducted five ML prediction model, which contains support vector machine (SVM), logistic regression (LR), random forest (RF), multilayer perceptron (MLP), K-nearest neighbor (KNN) and extreme gradient boosting (XGBoost) for the prediction of POP within 30 days in aSAH patients after surgical treatment. The best model will be verified in the MIMIC-IV database. To supply clinical basis for the prevention and therapy of POP (18).

## Materials and methods

### Study population

Data for aged more than 18 years aSAH patients treated with intracranial aneurysm embolization or aneurysm clipping in the First Affiliated Hospital of Wenzhou Medical University from 1 June 2017 to 4 February 2022 were retrospectively collected. Patients will be included in this study if they meet the following requirements: (1) aged 18 years or older, who suffered their first SAH ever, admitted to our hospital within 24 h of symptom onset; (2) All aSAH patients should be confirmed by computed tomographic (CT), computed tomographic angiography (CTA) and digital subtraction angiography (DSA); (3) endovascular coiling of the aneurysm was performed; and (4) The definition of POP in this study refers to lower respiratory tract infections that occur within 30 days after endovascular coiling procedure in our hospital and the diagnosis of POP should follow modified Centers for Disease Control and Prevention (CDC) criteria, which was characterized by the following criteria: (1) A probable case of POP cannot be diagnosed based solely on the admission or follow-up chest x-ray, and it cannot be attributed to another diagnosis. (2) A proven case of POP is confirmed when there is a documented change in diagnosis observed on at least one chest x-ray image. For the purpose of this study, patients meeting the modified CDC criteria for probable/proven pneumonia were considered as cases, while those with pre-existing pneumonia prior to admission were excluded from analysis (19). A total of 893 patients met the above conditions, among which 187 patients were excluded for the following reasons: (1) 115 patients with other potential causes of SAH (96 patients with arteriovenous malformation, 10 patients with craniocerebral trauma, and 9 patients with hypertensive intracerebral hemorrhage); (2) 14 patient with history of malignant tumors, severe heart, hepatic or renal failure; (3) 7 patient previous use of antibiotics, systemic glucocorticoids, immunosuppressive agents, or immunotherapy within 1 month before admission; (4) proven POP had a confirmed change in diagnosis on at least one image of the chest x-ray, and 35 patients with pneumonia before admission were excluded (2, 19); (5) 14 aSAH patients who died within 24 h after surgery or lack complete case records were excluded. Finally, 706 patients were included in the internal cohort.

**Figure 1:**
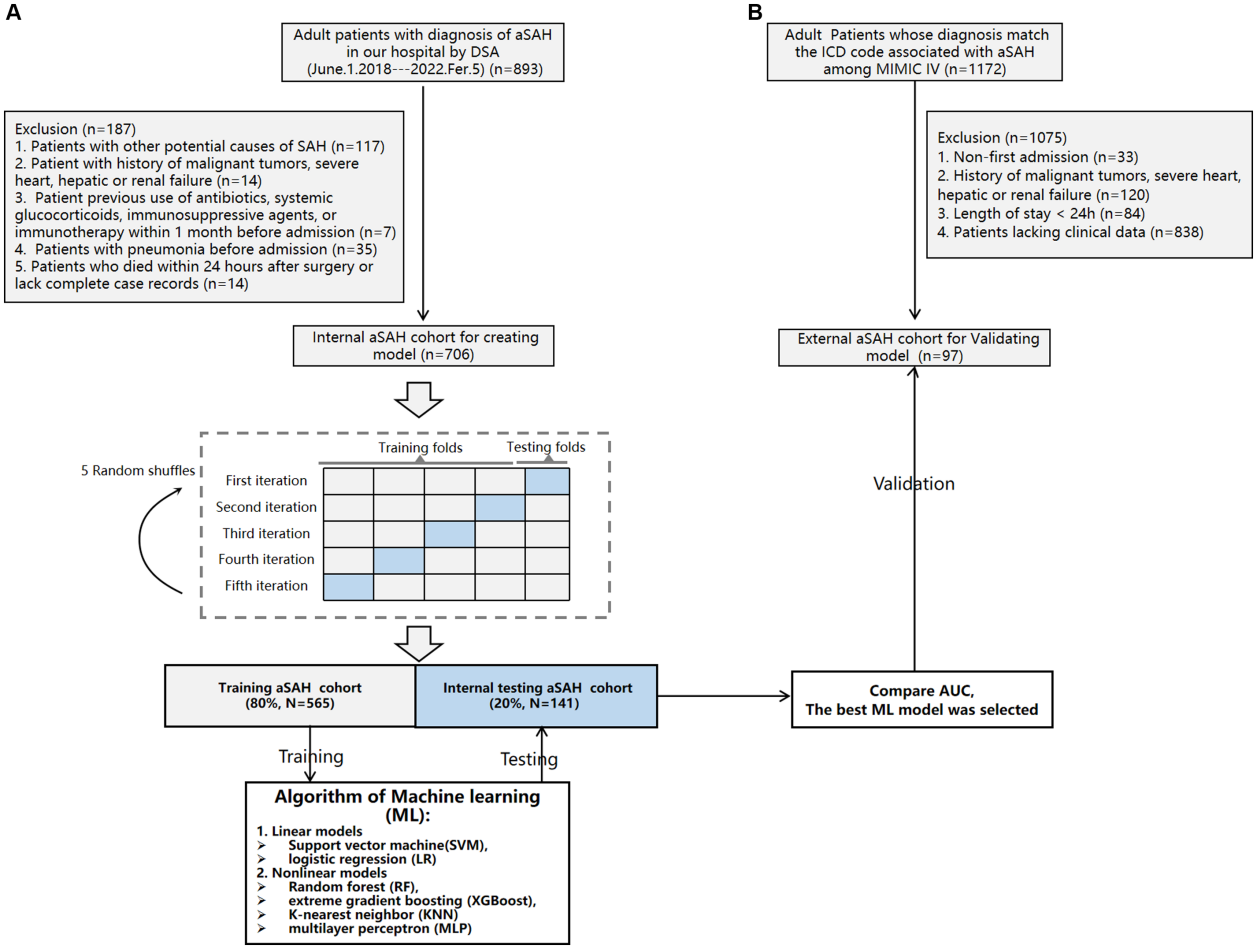
Flowchart of the internal cohort **(A)** and external cohort **(B)**.

The external cohort was extracted from the Medical Information Mart for Intensive Care IV (MIMIC-IV) (version 2.2) database, derived from a large, freely accessible critical care database comprising 299,712 patients who were admitted to the ICU or the emergency department of Beth Israel Deaconess Medical Center between 2008 and 2019^1^ (11). For the external cohort, individuals who met the following criteria were included: (1) those aged 18 years or older and (2) those with a diagnosis matching the International Classification of Diseases (ICD) code associated with aSAH among MIMIC-IV (ICD-9 code 430, and ICD-10 codes I60 to I609). A total of 1,172 patients fulfilled these conditions. Exclusion criteria consisted of: (1) non-first admission cases (*n* = 33); (2) history of malignant tumors, severe heart, hepatic or renal failure (*n* = 120); (3) length of stay less than 24 h in hospitalization (*n* = 84); and (4) patients without clinical data available for analysis (*n* = 838). Ultimately, a validation set comprising of 97 patients was selected from this group to validate the ML model. Figure 1 provides an overview of this process.

### Variable selection

Collected variables included: (1) patients demographics (age, gender, history of smoking, alcoholics); (2) GCS, Hunt-Hess grades, modified Fisher (mFS) grade and WFNS grade on admission; (3) past medical history (hypertension, diabetes mellitus), coronary heart disease (CHD), chronic obstructive pulmonary disease (COPD), Stent assisted endovascular treatment (EVT), with craniotomy; (4) aneurysm location [anterior communicating artery (ACoA), internal carotid artery (ICA), middle cerebral artery (MCA), posterior communicating artery (PCoA), vertebrobasilar artery (VBA)]; (5) laboratory results were obtained within 24 h after admission in the context of a first examination [albumin, hemoglobin, neutrophils, monocytes, lymphocytes, uric acid, total cholesterol, triglycerides, neutrophils, monocytes, mean corpuscular volumec-reactive (MCV), CRP], and the composite index calculated by these laboratory results NLR = Neutrophil counts (*10^9^ /L)/Lymphocyte count (*10^9^ /L), PNI = Albumin (g/L) +10*Lymphocyte count (*10^9^ /L), NAR = Neutrophil counts (*10^9^ /L)/Albumin (g/L), CAR = CRP (mg/L) / Albumin (g/L), D-Dimer/Albumin Ratio (DAR) = D-dimer level (μg/mL)/Albumin (g/L).

The developed ML model of risk factors associated with POP in aSAH patients is relied on statistically significant results obtained through LASSO regression and multivariate logistic regression analysis (*p* < 0.05) (20).

### ML model construction

A total of 706 patients with aSAH from our center were enrolled. We randomly divided the patients as training cohort (*N* = 565) and validation cohort (*N* = 141) according to a ratio of 80–20%. The training cohort was utilized to develop Linear models (logistic regression (LR), support vector machine (SVM)) and Nonlinear models [XGBoost, k-nearest neighbor (KNN), random forest (RF), and multilayer perceptron (MLP)] (21–23). XGBoost model was constructed using the xgboost package.^2^ The remaining five models were established via Scikit-learn package.^3^ To develop an unbiased assessment of model performance, we performed 5 random shuffles of 5-fold cross-validation, as shown in Figure 1. Each iteration used a different stratified fold for model evaluation, and the remaining folds were used for model training (24). Subsequently, we recorded Area Under The Curve (AUC) to compare each ML models. Data processing and the ML process are summarized in Figure 1.

After the model was established, the SHapley Additive exPlanations (SHAP) package in Python was used to explain the model by analyzing two cases. The SHAP package interpreted the output of the machine learning model using a game-theoretic approach (25). SHA*p* values quantify the association of a variable with the outcome of a single patient, and the mean absolute SHAP value across all patients is reported as the SHAP value of avariable (26).

### Statistical analysis

Continuous variables are represented in terms of mean and standard deviation (SD), while categorical variables are expressed in terms of frequency and percentage. Normally distributed and non-normally distributed variables were presented in the form of mean ± standard deviation and median (interquartile range). LASSO regression model was used to deal with collinearity of candidate variables, and the optimal predictive variable was selected (27). A multivariate logistic regression analysis was generated using predictors selected from the lasso analysis. Te features were represented by odds ratio (OR) and 95% confidence interval (CI). A two-tailed P value <0.05 was considered statistically significant. The stability of the columns in the validation queue is calculated using 1,000 boot replicas and a relative corrected C-index is calculated. All Statistical analyses were performed using R version 3.6.3 and python version 3.7.

## Results

### Demographic characteristics

Table 1 shows the clinical characteristics of the study population. A total of 706 aSAH patients were included in this study, which were divided into a training cohort (*N* = 565) and a validation cohort (*N* = 141). The number of patients with POP were 106 (16%) and 27 (12%) in training and testing cohorts, and men comprised 190 (34%) and 54 (38%) patients in the two groups, respectively. The median age in training cohort and testing cohorts were 55 and 59 years. The baseline data exhibited a high degree of consistency between the two groups (*p* > 0.05).

**Table 1:** Characteristics of the study population.

Variable	Total (*n* = 706)	Training cohort (*n* = 565)	Testing cohort (*n* = 141)	*p*
Age, median [IQR]	56.00 [49.00, 66.00]	55.00 [48.00, 66.00]	59.00 [51.00, 66.00]	0.090
LOS, median [IQR]	14.00 [10.00, 19.00]	14.00 [10.00, 20.00]	13.00 [9.00, 19.00]	0.140
Gender (male), n (%)	244 (34.56)	190 (33.63)	54 (38.30)	0.297
Alcoholics, n (%)	90 (12.75)	78 (13.81)	12 (8.51)	0.092
Smoking history, n (%)	82 (11.61)	68 (12.04)	14 (9.93)	0.485
Hypertension, n (%)	353 (50.00)	288 (50.97)	65 (46.10)	0.300
CHD, n (%)	21 (2.97)	16 (2.83)	5 (3.55)	0.655
Diabetes, n (%)	51 (7.22)	41 (7.26)	10 (7.09)	0.946
COPD, n (%)	5 (0.71)	4 (0.71)	1 (0.71)	0.999
DCI, n (%)	41 (5.81)	36 (6.37)	5 (3.55)	0.199
Timing to DCI (day), mean (±SD)	6.1 ± 1.9	6.0 ± 2.0	6.3 ± 2.0	0.768
POP, n (%)	106 (15.01)	89 (15.75)	17 (12.06)	0.272
VBA aneurysm, n (%)	58 (8.22)	38 (6.73)	20 (14.18)	0.004
MCA aneurysm, n (%)	111 (15.72)	90 (15.93)	21 (14.89)	0.762
ICA aneurysm, n (%)	204 (28.90)	167 (29.56)	37 (26.24)	0.437
PCoA aneurysm, n (%)	149 (21.10)	119 (21.06)	30 (21.28)	0.955
ACoA aneurysm, n (%)	229 (32.44)	176 (31.15)	53 (37.59)	0.144
With Craniotomy, n (%)	120 (17.00)	94 (16.64)	26 (18.44)	0.610
Stent-assisted EVT, n (%)	303 (42.92)	245 (43.36)	58 (41.13)	0.633
GCS, median [IQR]	15.00 [14.00, 15.00]	15.00 [14.00, 15.00]	15.00 [15.00, 15.00]	0.603
Hunt-Hess grades, median [IQR]	2.00 [2.00, 2.00]	2.00 [2.00, 2.00]	2.00 [2.00, 2.00]	0.523
WFNS, median [IQR]	1.00 [1.00, 2.00]	1.00 [1.00, 2.00]	1.00 [1.00, 1.00]	0.632
mFS, median [IQR]	2.00 [1.00, 3.00]	2.00 [1.00, 3.00]	1.00 [1.00, 2.00]	0.103
MVT, day, median [IQR]	0.00 [0.00, 0.00]	0.00 [0.00, 0.00]	0.00 [0.00, 0.00]	0.104
Albumin (g/L), median [IQR]	38.90 [36.00, 41.50]	39.00 [36.00, 41.50]	38.70 [36.00, 41.40]	0.628
Glucose (mmlo/L), median [IQR]	6.40 [5.40, 7.60]	6.40 [5.40, 7.60]	6.60 [5.20, 7.80]	0.665
Triglyceride (μmlo/L), median [IQR]	1.07 [0.82, 1.64]	1.08 [0.82, 1.69]	1.05 [0.81, 1.50]	0.388
Uric acid (μmlo/L), median [IQR]	242.00 [176.00, 301.00]	241.00 [176.00, 301.00]	249.00 [174.00, 302.00]	0.414
Total cholesterol (μmlo/L), median [IQR]	4.87 [4.30, 5.64]	4.90 [4.30, 5.66]	4.83 [4.02, 5.59]	0.197
Neutrophil counts (*10^9^/L), median [IQR]	9.82 [6.97, 12.70]	9.60 [6.96, 12.54]	10.05 [7.00, 12.70]	0.334
Monocyte count (*10^9^/L), median [IQR]	0.47 [0.30, 0.70]	0.47 [0.31, 0.71]	0.47 [0.27, 0.70]	0.319
Lymphocyte count (*10^9^ /L), median [IQR]	1.13 [0.86, 1.54]	1.14 [0.88, 1.55]	1.08 [0.72, 1.49]	0.258
Hemoglobin (g/L), median [IQR]	132.00 [121.00, 142.00]	132.00 [120.00, 142.00]	131.00 [121.00, 141.00]	0.574
MCV, (fl), median [IQR]	89.50 [86.40, 92.60]	90.00 [86.80, 92.70]	88.70 [86.00, 91.00]	0.003
Blood platelet count (*10^9^/L), median [IQR]	213.00 [175.00, 257.00]	214.00 [175.00, 258.00]	211.00 [174.00, 254.00]	0.584
CRP (mg/L), median [IQR]	6.00 [2.60, 15.70]	6.00 [2.90, 15.70]	5.60 [1.30, 17.40]	0.298
D-dimer level (μg/mL), median [IQR]	1.14 [0.55, 2.48]	1.15 [0.54, 2.56]	1.10 [0.56, 2.19]	0.196
PNI, median [IQR]	45.05 [41.80, 48.30]	45.10 [41.80, 48.50]	44.90 [41.80, 47.60]	0.518
NAR, median [IQR]	0.25 [0.19, 0.33]	0.25 [0.19, 0.32]	0.25 [0.20, 0.34]	0.279
CAR, median [IQR]	0.16 [0.07, 0.42]	0.17 [0.07, 0.41]	0.14 [0.04, 0.45]	0.296
NLR, median [IQR]	8.55 [5.16, 13.75]	8.48 [5.11, 13.75]	8.74 [6.22, 13.75]	0.337
DAR, median [IQR]	2.96 [1.42, 6.49]	3.00 [1.38, 6.70]	2.74 [1.47, 6.05]	0.209

LOS, length of stay; ACoA, anterior communicating artery; PCoA, posterior communicating artery; ICA, Internal Carotid Artery; MCA, middle cerebral artery; VBA, vertebrobasilar aneurysm; POP, postoperative pneumonia; CRP, C-reactive protein; MCV, Mean Corpuscular Volume; GCS, Glasgow Coma Scale; WFNS, World Federation of Neurosurgical Societies; Stent-assisted EVT, Stent assisted endovascular treatment; DCI, delayed cerebral ischemia; PNI, prognostic nutrition index; NAR, neutrophil-to-albumin Ratio; NLR, neutrophil-to-lymphocyte ratio; CAR, C-reactive protein-to-albumin ratio; DAR, D-Dimer-to-Albumin Ratio; MVT, mechanical ventilation time.

### Feature selection

**Figure 2:**
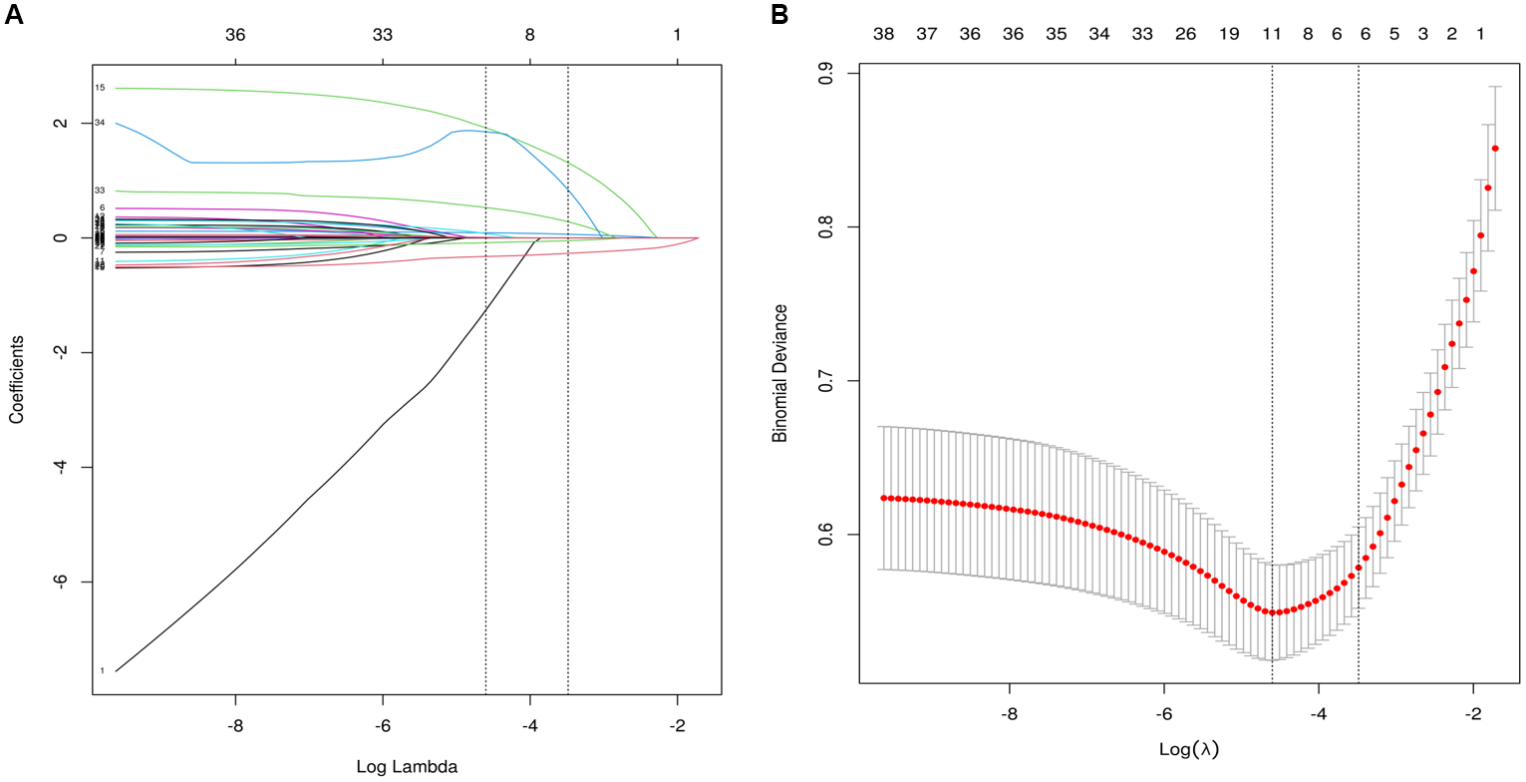
Perioperative variable selection using a LASSO logistic regression model. **(A)** The minimum criteria (lambda.min) and 1 SE of the minimum criteria (lambda. 1se) were used to depict the optimal values with dotted vertical lines. **(B)** LASSO coefficient profile of 39 variables. The coefficient profile is plotted according to the logarithmic sequence. To determine the optimal predictors of the model, five-fold cross-validation with minimum criteria was used, resulting in seven features with nonzero coefficients.

As partially relevant or less important features may negative affect performance of machine learning models, we performed feature selection by using LASSO regression. 39 variables with missing values <20% was extracted after interpolation, and 6 potential predictors were finally screened from the LASSO regression analysis (Figure 2). We included these 6 variables in multi-factor logistic regression and found that all 6 variables were statistically significant (Table 2). These 6 features were listed as follows: GCS (OR, 0.72; 95% CI, 0.58–0.73; *p* < 0.001), MVT (OR, 1.11; 95% CI, 1.03–1.21; *p* = 0.01), Albumin (OR, 0.90; 95% CI, 0.85–0.96; *p* < 0.001), NAR (OR, 55.23; 95% CI, 4.74–657.27; *p* < 0.001), CAR (OR, 2.61; 95% CI, 1.59–4.29; *p* < 0.001), Smoking history (OR, 8.37; 95% CI, 3.74–18.84; *p* < 0.001).

**Table 2:** Multivariable Logistic Regression Model for Predicting postoperative pneumonia in aSAH patients.

Predictor	β	SE	*p* value	Odds ratio (95% CI)
Intercept	5.14	2.13	0.460	196.4 (10.72–4054.43)
GCS, per score	−0.42	0.06	<0.001	0.72 (0.58–0.73)
MVT, per day	0.11	0.04	0.010	1.11 (1.03–1.21)
Albumin, g/L	−0.10	0.03	<0.001	0.90 (0.85–0.96)
NAR, per score	4.01	1.25	<0.001	55.23 (4.74–657.27)
CAR, per score	0.96	0.25	<0.001	2.61 (1.59–4.29)
Smoking history (yes vs. no)	2.12	0.41	<0.001	8.37 (3.74–18.84)

MVT, mechanical ventilation time; GCS, Glasgow Coma Scale; NAR, neutrophil-to-albumin Ratio; CAR, CRP-to-albumin ratio.

### Machine learning model performance

**Figure 3:**
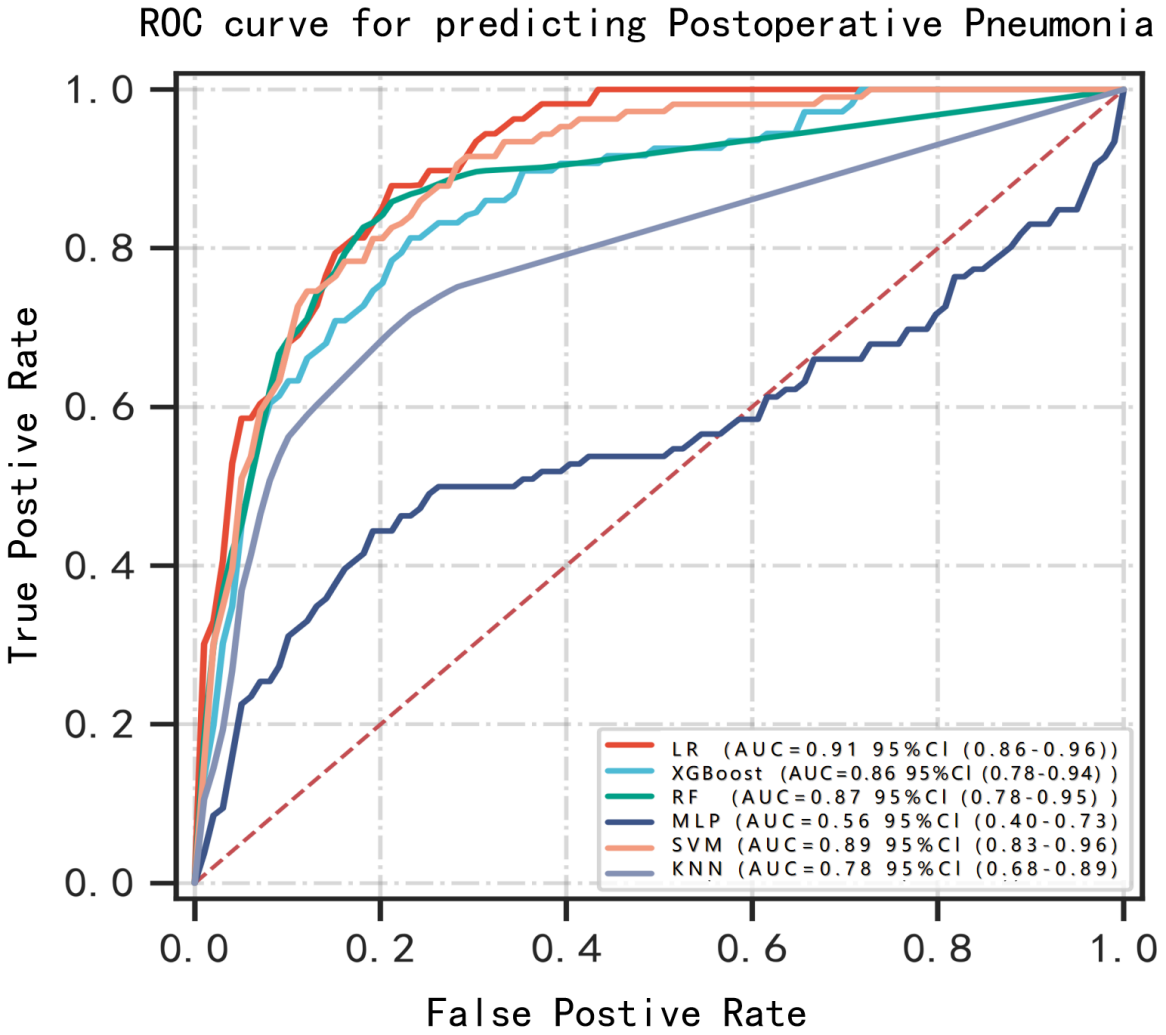
ROC curves for prediction of postoperative pneumonia (POP) on the test data set. Greater AUC shows higher discriminative ability of the model.

**Figure 4:**
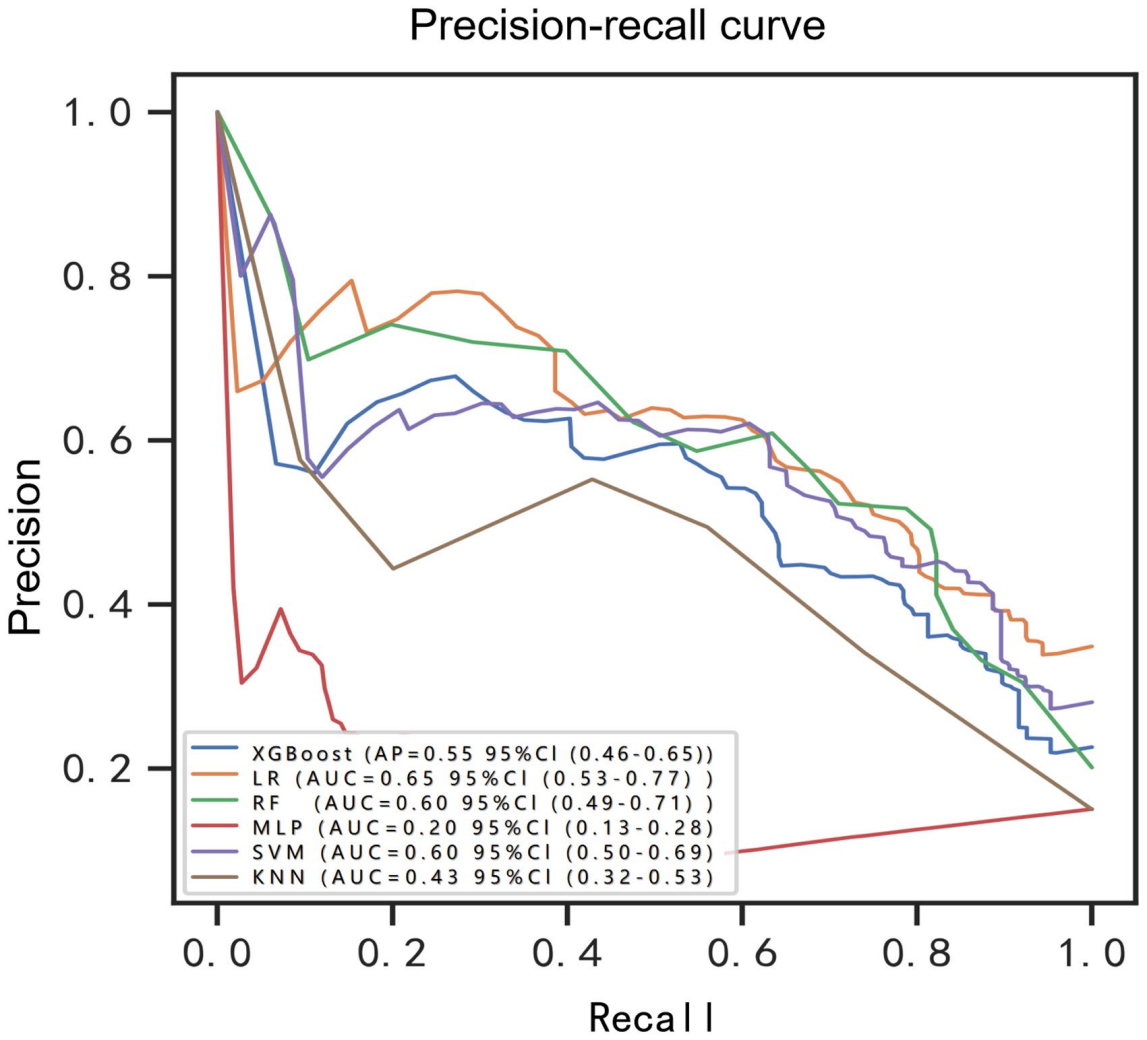
Precision-recall curve for prediction of postoperative pulmonary risk. BRF, balanced random forest; CI, confidence interval; LR, logistic regression; XGBoost, extreme gradient boosting; RF, random forest; MLP, multilayer perceptron; SVM, support vector machine; KNN, K-nearest neighbor.

Using the six features obtained by screening, we developed six machine learning models, including LR, SVM, RF, MLP, XGBoost, and KNN. Supplementary Table S1 and Figure 3 showed the best hyperparameter combination for each model and their AUCs in predicting POP. Their performance for prediction of POP was assessed (Table 3). The AUC values of KNN (0.78) and MLP (0.56) were relatively lower than LR (0.91), SVM (0.89), RF (0.87), XGBoost (0.86). Among them, LR exhibited the best performance for the prediction of POP risk. As the primary metric, the AUC for LR was 0.91 (95% confidence interval: 0.86–0.96). LR also exhibited the best performance based on the average precision of the precision-recall curve (0.65). The average precisions of the precision-recall curve for the remaining models are summarized in Figure 4. The remaining metrics are summarized in Table 3.

**Table 3:** Performance of the six ML models in the testing set.

ML models	AUC (95%CI)	Accuracy (95% CI)	Sensitivity (95% CI)	Specificity (95% CI)
LR	0.91 (0.86–0.96)	0.81 (0.78–0.84)	0.94 (0.91–0.98)	0.78 (0.69–0.87)
XGBoost	0.86 (0.78–0.94)	0.87 (0.85–0.88)	0.81 (0.67–0.96)	0.81 (0.73–0.89)
RF	0.87 (0.78–0.95)	0.87 (0.84–0.90)	0.83 (0.76–0.90)	0.85 (0.79–0.90)
MLP	0.56 (0.40–0.73)	0.76 (0.74–0.78)	0.48 (0.39–0.57)	0.80 (0.76–0.85)
SVM	0.89 (0.83–0.96)	0.79 (0.77–0.81)	0.87 (0.85–0.89)	0.81 (0.74–0.88)
KNN	0.78 (0.68–0.89)	0.85 (0.83–0.88)	0.70 (0.57–0.83)	0.81 (0.72–0.91)

LR, logistic regression; XGBoost, extreme gradient boosting; RF, random forest; MLP, multilayer perceptron; SVM, support vector machine; KNN, K-nearest neighbor.

### Application of the model

**Figure 5:**
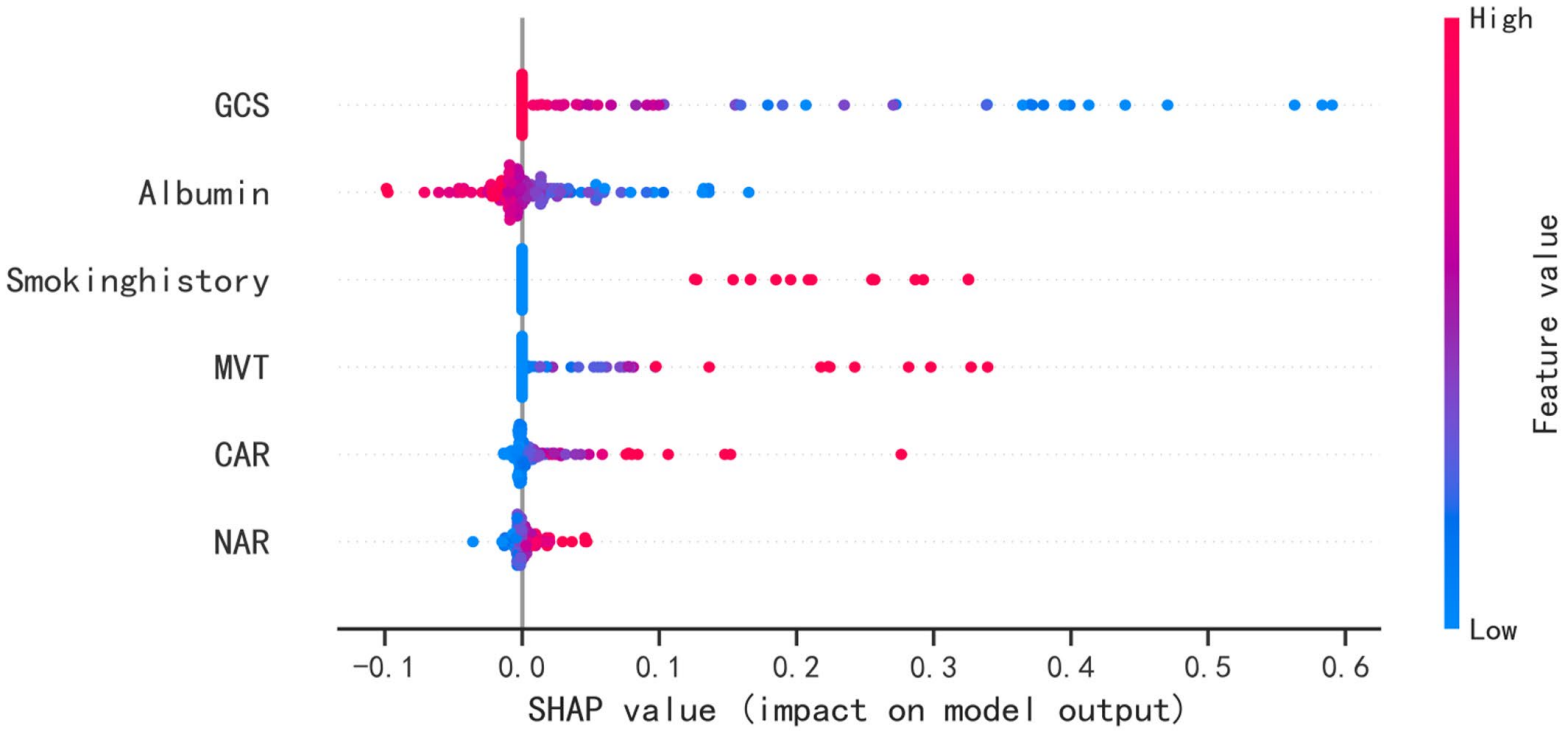
SHAP analysis of the proposed model on the testing set. This figure described data from the testing set, with each point representing one patient. The color represents the value of the variable; red represents the larger value; blue represents the smaller value. The horizontal coordinates represent a positive or negative correlation with transfusion risk, with a positive value indicating a risk of POP and a negative value indicating no risk for POP. The absolute value of the horizontal coordinate indicates the degree of influence; the greater the absolute value of the horizontal coordinate, the greater the degree of influence.

**Figure 6:**
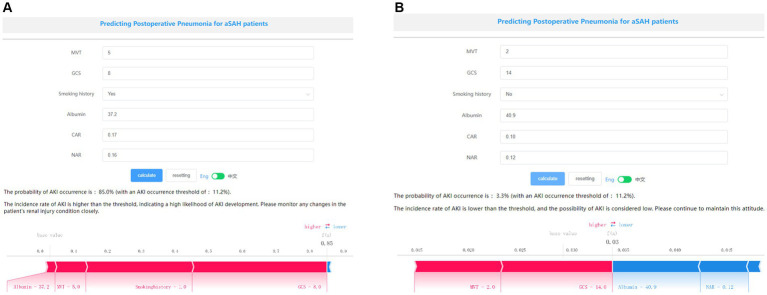
Examples of website usage. Entering the input value determined the transfusion requirements and displayed how each value contributed to the prediction. **(A)** Example 1 has a high probability of POP, the probability of POP is 85.0%, and **(B)** Example 2 has a low probability of POP, the probability of POP is 3.3%.

The SHAP package conducted a comprehensive analysis of training set, showing the impact of each variable on predicting POP (Figure 5). The preoperative and postoperative information of a patient was input into the model: with mechanical ventilation 5 days, the GCS score at admission was 8, albumin level 37.2 g/L, with smoke history, CAR 0.17, NAR 0.16. The model analyzed that the risk of POP in this patient was 85.0%, indicating that the probability of POP for the patients was high, and the implementation of preventive treatments against POP should be prioritized (Figure 6A). The preoperative and postoperative information of another patient was input into the model: with mechanical ventilation 2 days, the GCS score at admission was 14, albumin level 40.9 g/L, with no smoke history, CAR 0.10, NAR 0.12. The model analyzed that the risk of POP in this patient was 3.3%, which indicated a low probability of POP occurrence in the patients and the occurrence of POP in the patient does not require significant attention (Figure 6B). Furthermore, a website was established for clinicians to use the proposed LR model.^4^

### External data validation

**Figure 7:**
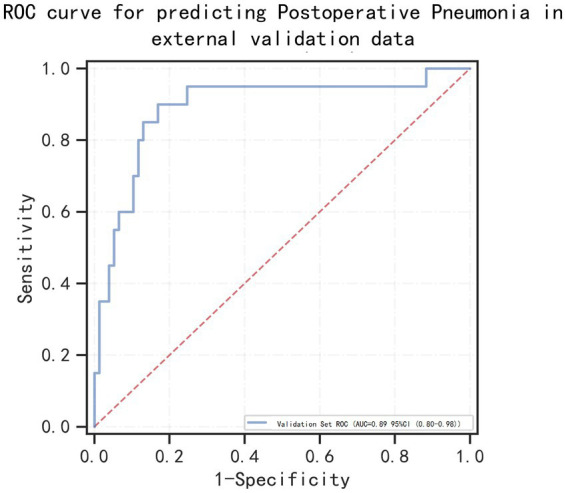
Receiver operating characteristic (ROC) curves for predicting postoperative pneumonia in aneurysmal subarachnoid hemorrhage patients in the external valication set.

To further confirmed the applicability of our model, we performed external validation using data from 97 patients with aSAH with MIME-IV. Figure 7 showed that the AUC of the model in the external data is 0.89 (95% confidence interval: 0.80–0.98), indicating that the model can still play a very good predictive performance in the external data.

## Discussion

Early detection of POP is critical for timely interventions to prevent the onset of the complication (21). Numerous studies have developed ML models to predict postoperative pulmonary complications. Jong Ho Kim et al. developed an ML model to predict POP in patients undergoing surgery (28). Peng et al. had successfully created and verified a deep-neural-network model based on combined natural language data and structured data to predict pulmonary complications in geriatric patients (29). However, there is no POP prediction ML model designed explicitly for aSAH patients. Therefore, it is urgent to establish a POP clinical prediction model for aSAH patients, which has good application value in clinical identification and decision-making.

We used ML to develop models for the prediction of POP for aSAH patients. Model training using data from 565 patients was followed by model testing using data from 141 patients. Six algorithms (LR, XGBoost, RF, MLP, SVM, KNN) were used to develop the models, whereas four metrics were used to evaluate their performances. LR exhibited the best overall performance, with a specificity of 78% and a sensitivity of 94% in predicting POP in aSAH patients. Besides, the AUC values of MLP and KNN were relatively lower than XGBoost, RF and SVM, whose accuracy and robustness might be attributed to their nature of integrating multiple base classifiers or learners (21). In addition, two examples were used to visualize how the LR model could predict POP and determine the relative importance of each variable for the clinician. With millions of POP taking place each year, the findings could help surgeons perform the management of postoperative pulmonary infection, such as the consideration of ventilator use time and tracheotomy, the use of prophylactic antibiotics. Finally, In order to reflect the model has a extensive range of applications, LR model was also validated on external data (MIMIC-IV) and showed good predictive performance.

In addition to successfully constructing machine learning models for predicting patient outcomes, the selection of variables should also prioritize the clinical requirements. As widely acknowledge, feature selection is a crucial process in machine learning. Selecting the proper combination of features to achieve a balance between model performance and efficiency is difficult but of great significance (30). Previous ML models required many features to predict Prognosis of patients, which reduces its practicality (28). We identified this issue at an early stage, prompting us to initially conduct LASSO regression on 39 variables. This approach not only mitigated the problem of collinearity among variables, but also resulted in a reduction of included variables in the model to six. Therefore, our ML models incorporated 6 variables of GCS, smoking history, MVT, GCS, NAR, CAR as predictors. These variables can be easily collected in clinical practice and important implications in clinical practice.

Among these variables, CAR was identified for the first time as an independent risk factor for predicting the POP in patients with aSAH. Previously, Dingding Zhang et al. discovered that an elevated CAR was correlated with the WFNS grade and Glasgow Outcome Scale (GOS) after 3 months aSAH (31). Our study further confirmed the importance of CAR on the prognosis of aSAH, and we also found that CAR was a better predictor of POP than other composite indicators such as NLR, NAR, DAR, and PNI (Supplementary Figure S1). Although Xin Zhang and Manman Xu et al. had demonstrated that NAR, PNI, DAR, and NLR are independent risk factors for predicting POP separately (2, 5, 12, 13), if these factors are compared together, the AUC of CAR and NAR was better than that of NLR, DAR and PNI, which also makes CAR and NAR remain as predictors into our model, while other indicators were excluded due to lower AUC or too high correlation coefficient with CAR and NAR. Back to the nature of the CAR, many previous studies had demonstrated the role of CRP and albumin in predicting pneumonia. CRP, an acute-phase protein, is triggered by different cytokines in reaction to infection, ischemia, trauma, and other inflammatory circumstances (32). In a recent study, Ben Gaastra et al. also discovered that CRP serves as an independent prognostic indicator for outcome following aSAH. The incorporation of CRP into prognostic models enhances their predictive accuracy (33). Xinlong Ma et al. found early increase in blood CRP appears to correlate with poor functional outcome after aSAH (34). Such patients exhibit a protracted recovery period for cough, expectoration, and swallowing function, necessitating an extended bed rest duration and presenting an increased susceptibility to pulmonary infections. Among them, the level of albumin played a pivotal role in our model, as it not only functioned as an independent prognostic indicator but also contributed to the calculation of two crucial predictive indicators, CAR and NAR. We posit that the primary factor contributing to this phenomenon is the multifaceted impact of hypoalbuminemia on patients with aSAH. Hypoalbuminemia in patients with aSAH not only reduces the level of immune protein and obstructs the repair of the mucosal barrier, leading to increased susceptibility to infection, but also exacerbates brain edema after subarachnoid hemorrhage, ultimately worsening motor function impairment in patients (32, 35). Some recent experimental findings additionally indicate that albuminemia mediates its neuroprotection through neurovascular remodeling and reducing cerebral lesions (36–38). A pilot study revealed that 1.25 g/kg/day albumin treatment was safe in SAH patients and might produce a better outcome (37). The clinicians should therefore prioritize the monitoring of hypoalbuminemia in aSAH patients, and consider implementing albumin supplementation as a preventive measure against infection. There are few clinical studies in this area, and further research is warranted to establish more advanced clinical diagnostic and treatment protocols.

In addition, we also note that Xiao Jin et al. constructed a nomogram model for predicting POP in aSAH patients (11). All of our models use MIMIC-IV data as our external data to verify model efficacy. Xiao Jin’s nomogram model had a validation performance of AUC 0.85 on external data, slightly lower than our LR model AUC 0.89. This is further evidence that, ML can offer unique perspective on the patient’s condition and can serve as a decision support tool in the management of aSAH. However, clinical judgment is necessary to interpret the ML results and implement a corresponding plan of action (17). For example, in the process of modeling, because our database only had 5 patients with COPD, and these patients had mild intracerebral hemorrhage, and none of them had POP, COPD was not included as an important predictor in the process of modeling. However, according to clinical experience, COPD patients are extremely prone to pulmonary infection. Therefore, the knowledge and experience of doctors are essential for the prediction of POP in aSAH patients with special conditions.

This study had several limitations. First, although the ML models have been validated in another database, the ML models are developed on the basis of a single-center cohort study, and future multi-center study will be needed for external validation. Furthermore, it should be noted that this study was conducted in a retrospective manner, which may have introduced collection and entry biases as well as residual confounding factors.” Third, in the case of external cohort inclusion, determining whether patients have received surgical treatment is challenging due to insufficient data availability. Hence, surgery was not considered as a prerequisite for enrolling an external cohort (11). In addition, it poses a significant challenge to monitor the occurrence of POP due to premature discharge resulting from severe illness or financial constraints, making it arduous to assess the likelihood of these patients developing POP. This limitation is equally applicable when utilizing the MIMIC-IV database, which solely documents the presence or absence of POP during hospitalization. Nonetheless, we believe that any potential error stemming from this constraint remains within manageable boundaries, as demonstrated in Xiao Jin et al.’s study (11). Last but not least, two or more machine learning algorithms can be synthesized to further improve predictive accuracy. Such a process is referred to as ensemble modeling, and it has been used broadly in various industries (39). However, because the authors of this paper were unfamiliar with this method, they did not dare to operate ensemble modeling arbitrarily. In the future, we will pay more attention to improving the prediction performance of clinical models by ensemble learning algorithms.

## Conclusion

Our study has successfully established six novel ML models to predict POP among aSAH patients. Of these, the LR model has demonstrated overall best performance. Furthermore, an online prediction tool based on the LR model was developed to identify patients at high risk for POP after aSAH and facilitate timely interventions.

## Data availability statement

The original contributions presented in the study are included in the article/Supplementary material, further inquiries can be directed to the corresponding author.

## Ethics statement

The studies involving humans were approved by the Ethics Committee in Clinical Research (ECCR) of the First Affiliated Hospital of Wenzhou Medical University. The studies were conducted in accordance with the local legislation and institutional requirements. Written informed consent for participation was not required from the participants or the participants’ legal guardians/next of kin in accordance with the national legislation and institutional requirements.

## Author contributions

XL: Writing – original draft, Writing – review & editing. CZ: Writing – original draft, Writing – review & editing. JW: Formal analysis, Data curation, Methodology, Writing – original draft, Writing – review & editing. CY: Writing – original draft, Writing – review & editing. JZ: —. QicZ: Writing – original draft, Writing – review & editing.

## Footnotes

^1^ https://mimic.physionet.org/

^2^ https://xgboost.readthedocs.io/en/latest/python/index.html

^3^ https://github.com/scikit-learn/scikit-learn

^4^ https://www.xsmartanalysis.com/model/list/predict/model/html?mid=9730&symbol=4170wQvi00hz43760JQ1
