# Supplementary material for: Development and performance assessment of novel machine learning models for predicting postoperative pneumonia in aneurysmal subarachnoid hemorrhage patients: external validation in MIMIC-IV
Source: Front Neurol. 2024 Apr 15;15:1341252. doi: 10.3389/fneur.2024.1341252 (PMC11056519; doi:10.3389/fneur.2024.1341252)
Supplement: Supplementary file 1 [file Table_1.DOC]

**Supplementary information**

**Table S1. Hyperparamter combinations for each machine learning models and their corresponding test set AUC**

|  | **Hyperparameters Set** | **Optimal Hyperparameters** | AUC |
| --- | --- | --- | --- |
| LR | C: 10.0 max iter: 100 penalty: l2 tol: 0.0001 | L1 Regularization term: 10.0 | 0.915 |
| SVM | Kernel:{Linear, Polynomial, Radial Basis, Sigmoid} Regularization term: {0,1,2,3,4,5} | Kernel: Radial Basis Regularization term: 1 | 0.896 |
| RF | Criterion: gini max_depth: None min impurity decrease: 0.0 n estimators: 100 | Number of estimators: 100 Maximum depth: None | 0.994 |
| KNN | Number of neighbors: 6 weights: distance | Number of estimators: 500 Learning rate: 0.05 | 0.995 |
| MLP | activation: logistic hidden layer sizes: (30, 30) max iter: 10 | L2 Regularization term: 0.1 | 0.671 |
| XGB | Number of estimators: {50,100,200,500} Learning rate: {0.1,0.05,0.01} Maximum depth: {3,4,5,6} | objective: binary:logistic learning rate: 0.3 max depth: 8 min child weight: 2 L2 Regularization term: 0.5 | 0.992 |

Note: LR, logistic regression; SVM, support vector machine; RF, random forest; MLP, multilayer perceptron; KNN, K-nearest neighbor;; XGB, extreme gradient boosting. AUC score measured the model performance on testing set.

**Table S2.** TRIPOD checklist

| Section and topic | Item No | Checklist item |
| --- | --- | --- |
| Title |  |  |
| Title | 1 | [Development and Performance Assessment of Novel Machine learning Models for Predicting](https://pubmed.ncbi.nlm.nih.gov/34921550/) Postoperative Pneumonia in Aneurysmal Subarachnoid Hemorrhage Patients: [External validation in MIMIC-IV.](https://pubmed.ncbi.nlm.nih.gov/35526587/) |
| Background |  |  |
| Objectives | 2 | Postoperative pneumonia (POP) is one of the primary complications after aneurysmal subarachnoid hemorrhage (aSAH) and is associated with postoperative mortality, extended hospital stay, and increased medical fee. Early identification of pneumonia and more aggressive treatment can improve patient outcomes. We aimed to develop a model to predict POP in aSAH patients using machine learning (ML) methods. |
| Methods |  |  |
| Study eligibility criteria | 3 | Ethical review and approval was not required for the study on human participants in accordance with the local legislation and institutional requirements. Written informed consent for participation was not required for this study in accordance with the national legislation and the institutional requirements. |
| Information sources | 4 | Internal cohort: First Affiliated Hospital of Wenzhou Medical University from 1 June 2017 to 4 February 2022.  External cohort: Medical Information Mart for Intensive Care IV (MIMIC-IV) (version 2.2) database, derived from a large, freely accessible critical care database comprising 299,712 patients who were admitted to the ICU or the emergency department of Beth Israel Deaconess Medical Center between 2008 and 2019 (https://mimic.physionet.org/). |
| Risk of bias and applicability | 5 | Although the ML models have been validated in another database, the ML models are developed on the basis of a single-center cohort study. Patients will be included in this study if they meet the following requirements: (1) aged 18 years or older, who suffered their first SAH ever, admitted to our hospital within 24 h of symptom onset; (2) All aSAH patients should be confirmed by computed tomographic (CT), computed tomographic angiography (CTA) and digital subtraction angiography (DSA); (3) endovascular coiling of the aneurysm was performed. (4) The diagnosis of POP should follow modified Centers for Disease Control and Prevention (CDC) criteria: (1) A probable POP could not be diagnosed based on the admission or the follow-up chest x-ray, and it could neither be explained by another diagnosis |
| Synthesis methods | 6 | Each iteration used a different stratified fold for model evaluation, and the remaining folds were used for model training(24). Subsequently, we recorded Area Under The Curve (AUC) to compare each ML models. Data processing and the ML process are summarized in Figure 1.  After the model was established, the SHapley Additive exPlanations (SHAP) package in Python was used to explain the model by analyzing two cases. |
| Results |  |  |
| Included studies | 7 | This internal cohort study included 706 patients with aSAH undergoing intracranial aneurysm embolization or aneurysm clipping. The cohort was randomly split into a train set (80%) and a testing set (20%). Perioperative information was collected from participants to establish 6 machine learning models for predicting POP after surgical treatment. The area under the receiver operating characteristic curve (AUC), precision-recall curve were used to assess the accuracy, discriminative power, and clinical validity of the predictions. The final model was validated using an external validation set of 97 samples from the Medical Information Mart for Intensive Care IV (MIMIC-IV) database. |
| Results of syntheses | 8 | Using the six features obtained by screening, we developed six machine learning models, including LR, SVM, RF, MLP, XGBoost, and KNN. Supplementary Table S1 and Figure 3 showed the best hyperparameter combination for each model and their AUCs in predicting POP. Their performance for prediction of POP was assessed (Table 3). The AUC values of KNN (0.78) and MLP (0.56) were relatively lower than LR (0.91), SVM (0.89), RF (0.87), XGBoost (0.86). Among them, LR exhibited the best performance for the prediction of POP risk. As the primary metric, the AUC for LR was 0.91 (95% confidence interval: 0.86–0.96). LR also exhibited the best performance based on the average precision of the precision-recall curve (0.65). |
| Discussion |  |  |
| Limitations of evidence | 9 | First, although the ML models have been validated in another database, the ML models are developed on the basis of a single-center cohort study, and future multi-center study will be needed for external validation. Furthermore, it should be noted that this study was conducted in a retrospective manner, which may have introduced collection and entry biases as well as residual confounding factors." Third, in the case of external cohort inclusion, determining whether patients have received surgical treatment is challenging due to insufficient data availability. Hence, surgery was not considered as a prerequisite for enrolling an external cohor. |
| Interpretation | 10 | All of our models use MIMIC-IV data as our external data to verify model efficacy. Xiao Jin's nomogram model had a validation performance of AUC 0.85 on external data, slightly lower than our LR model AUC 0.89. This is further evidence that, ML can offer unique perspective on the patient's condition and can serve as a decision support tool in the management of aSAH. |
| Other |  |  |
| Funding | 11 | This study received funding from Wenzhou Science and Technology Bureau Major Project ZS2017007 and Key project of International Cooperation of National Natural Science Foundation 81820108011. |
| Registration | 12 | This was a retrospective study and no registration was required. |
