# Supplementary figures and images for: Development and performance assessment of novel machine learning models for predicting postoperative pneumonia in aneurysmal subarachnoid hemorrhage patients: external validation in MIMIC-IV
Source: Front Neurol. 2024 Apr 15;15:1341252. doi: 10.3389/fneur.2024.1341252 (PMC11056519; doi:10.3389/fneur.2024.1341252)

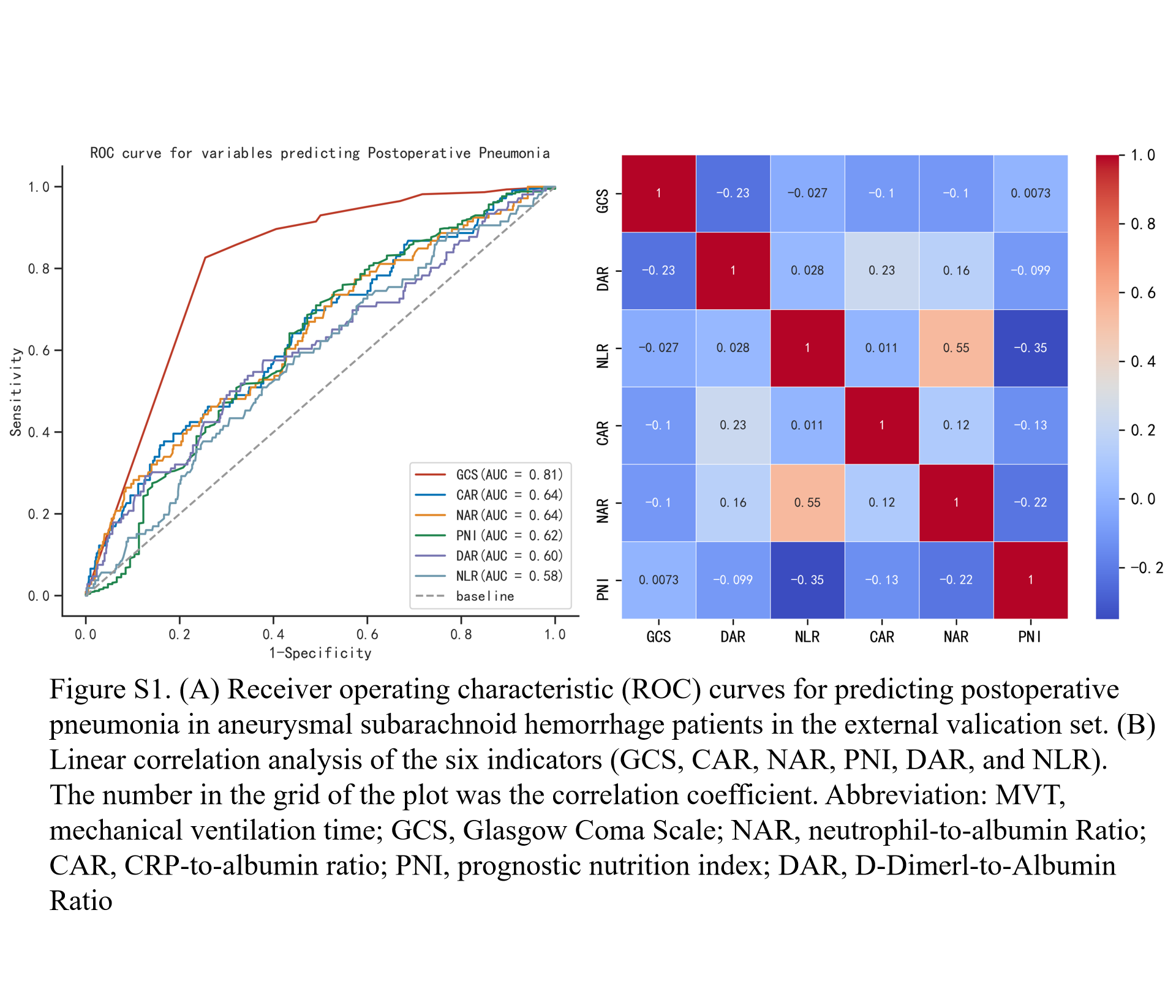

Supplement: Supplementary file 3 [file Image_1.TIF]
